# Cancer immunotherapy: from the lab to clinical applications—Potential impact on cancer centres’ organisation

**DOI:** 10.3332/ecancer.2016.691

**Published:** 2016-11-10

**Authors:** Linda Cairns, Sandrine Aspeslagh, Andrea Anichini, Jon Amund Kyte, Christian Blank, Paolo Ascierto, Nicolle Rekers, Per Thor Straten, Ahmad Awada

**Affiliations:** 1 European Institute of Oncology, Via Ripamonti, 435, 20141 Milan. Italy; 2 Gustave Roussy, 114 rue Edouard Vaillant, 94805 Villejuif cedex, France; 3 Fondazione IRCCS Istituto Nazionale dei Tumori, Via Giacomo Venezian, 1, 20133 Milano, Italy; 4 Oslo University Hospital, Postboks 4950 Nydalen, 0424 Oslo, Norway; 5 Netherlands Cancer Institute, Plesmanlaan 121, 1066 CX Amsterdam, The Netherlands; 6 Istituto Nazionale Tumori IRCCS ‘Fondazione G. Pascale’, Via Mariano Semmola, 80131 Napoli, Italy; 7 Maastricht University Medical Centre, Maastricht UMC+ P. Debyelaan 25 6229 HX Maastricht, The Netherlands; 8 University of Copenhagen Herlev Hospital, Herlev Ringvej 75, 2730 Herlev, Denmark; 9 Institut Jules Bordet, 1, rue Héger-Bordet, 1000 Brussels, Belgium

**Keywords:** immunotherapy, monoclonal antibodies, cancer vaccines

## Abstract

This report covers the Immunotherapy sessions of the 2016 Organisation of European Cancer Institutes (OECI) Oncology Days meeting, which was held on 15th–17th June 2016 in Brussels, Belgium. Immunotherapy is a potential cancer treatment that uses an individual’s immune system to fight the tumour. In recent years significant advances have been made in this field in the treatment of several advanced cancers. Cancer immunotherapies include monoclonal antibodies that are designed to attack a very specific part of the cancer cell and immune checkpoint inhibitors which are molecules that stimulate or block the inhibition of the immune system. Other cancer immunotherapies include vaccines and T cell infusions. This report will summarise some of the research that is going on in this field and will give us an update on where we are at present.

## Session 1: The laboratory background and current developments

**Sandrine Aspeslagh** from Gustave Roussy gave a presentation entitled ‘The experimental evidence for immune-mediated cell death during chemotherapy and radiotherapy’. She discussed the mechanisms and key importance of immunogenic cell death (ICD). She also discussed the mechanism of activation of the immune system which is triggered by the cellular stress generated by chemotherapy, and how it is thought to be essential for treatment response and induction of a durable post-chemotherapy immunity. Tumours which lack this mechanism respond less well to treatment and have a worse prognosis. Several types of conventional anti-cancer treatments such as chemotherapy and radiotherapy do elicit ICD and will lead to therapeutic success in immunocompetent but not immunodeficient hosts. This type of cell death will activate immune pathways involving calreticulin, ATP, and HMGB1 leading to a more adequate immune reaction towards the cancer cells and as such enhance the anti-tumour potential. In the era of immune checkpoint blockade it is of the utmost importance to understand these mechanisms in order to propose better rational combinatorial regimens for immune checkpoint resistant patients.

**Andrea Anichini** from the National Tumour Institute in Milan discussed ‘Technologies and current translational approaches in immunotherapy’. Over the past six years, results of several Phase I to III clinical trials of cancer immunotherapy targeting immune checkpoints (initially CTLA-4 and subsequently the PD-1/PD-L1 axis) have fostered the dawn of a new era in the treatment of several advanced cancers including melanoma, non-small cell lung cancer (NSCLC), urothelial cancer, renal cancer, head and neck squamous cell carcinoma, triple-negative breast cancer, gastric cancer, colorectal cancer, Merkel cell carcinomas, and Hodgkin’s lymphomas. In spite of these remarkable improvements in treatment of several advanced cancers, only a fraction of patients achieve clinical benefit from immunotherapy. The recent technological developments in the fields of pathology and immunopathology and of genomic and immunological analysis of neoplastic lesions have fostered the development of effective translational approaches that allow to understand the mechanisms of response and resistance to immunotherapy. It helps define which tumour subsets have a molecular and immunological profile that favours response to immunotherapy and also to identify predictive markers of response and resistance.

The first relevant development has been the introduction of the genomic analysis techniques that allows the profiling of all non-synonymous mutations existing in a tumour sample and to then infer the overall mutational burden and the neo-antigen load of each lesion. The relevance of the neo-antigen load in different solid tumours is a main predictive factor of response to immunotherapy.

A further remarkable technological improvement has been the introduction of fluorescence-based microscopy platforms that allow the development of quantitative and multi-parametric pathological analysis of tissues. These tools enable pathologists and immunologists to count single immune or stromal or neoplastic cells in the lesions and to understand the relative topological relationships in the light of the specific phenotypic and functional properties of each subset. The latter development is providing a quantitative means to assess key predictive parameters of response to immunotherapy as well as the immunoscore and the overall immune contexture for the pre- and post-therapy lesions.

Further translational approaches that are proving to be key tools of immunological investigation in the context of immunotherapy include techniques that allow the gene expression profiles to be measured in formalin-fixed, paraffin-embedded (FFPE)tissue. This enables one to understand whether a specific lesion has an active immune-related gene programme.

Finally, all existing experimental approaches, such as multiparametric flow cytometry and mass cytometry, that allow the *ex-vivo* analysis of function and antigen-specificity of T cells isolated from surgical samples help contribute to validate the potentially relevant neo-antigens that in each specific lesion are targeted by the immune system.

**Jon Amund Kyte** from Oslo University Hospital, Norway discussed ‘Adoptive cellular therapy approaches and early clinical studies’.

Adoptive cell therapy (ACT) with tumour-infiltrating lymphocytes in cancer therapy is already in use for the treatment of metastatic melanoma. More recently, modern gene engineering technology has opened for reprogramming the patient’s own T cells with tumour-targeting T cell receptors (TCRs) or chimeric antigen receptors (CARs). These T cells are then expanded to millions of tumour-specific ‘missiles’ and given back to the patient. A number of CAR T cell studies have shown remarkable clinical responses in heavily pre-treated patients with acute lymphoblastic leukaemia (80–90% complete responses), chronic lymphocytic leukaemia or B-cell lymphomas. The CARs represent optimised TCRs, comprising of an extracellular antigen binding single chain fragment variable (scFv) from a monoclonal antibody (mAb) and signalling domains from the TCR complex. Contrary to TCRs, the CAR binds in an HLA-independent manner and may be used across the entire patient population. While CARs can only target surface antigens, TCRs also recognise intracellular antigens which greatly broadens the spectrum available targets. ACT with redirected T cells has not yet shown efficacy against solid cancers. A number of approaches are currently investigated to address this limitation. Key areas of research include attempts to identify better target antigens, strategies to overcome established tolerance in solid tumours, and approaches for personalising the therapy by targeting patient-specific neo-antigens.

**Christian Blank** from the Netherlands Cancer Institute discussed the impact of cancer immunotherapy on clinical cancer care. Inhibitors of T cell checkpoints, such as PD-1 and CTLA-4, are showing clinical activity in a variety of human malignancies. The infusion of autologous tumour-infiltrating T cells has shown activity in melanoma and may also be of value in HPV positive cancers. Also the infusion of gene-modified T cells is showing clinical activity in particular for B cell malignancies. It is important to realise that these different immunotherapies remedy distinct ‘problems’ in tumour–immune interactions. For instance, PD-1 blockade is considered to primarily unleash the activity of an already tumour-resident T cell pool. In contrast, in addition to a possible effect at the tumour site, CTLA-4 blockade is thought to enhance tumour-specific T cell responses. Finally, gene-modified T cell therapies assume that the endogenous T cell compartment is insufficient, and a creation of a novel T cell compartment is the hurdle that needs to be taken to achieve tumour regression. There is also a question of what would be the most effective therapy for an individual patient?

Dr Blank outlined a framework to describe the interaction between cancer and immune system in individual cases, and that—on the basis of biomarker assays—may help predict which specific aspect of cancer–immune interaction should be the target for therapy. This Cancer Immunogram builds on two key observations. First, the outcome of cancer–immune interactions is based on a number of largely unrelated parameters, including aspects such as tumour foreignness and a series of T cell inhibitory mechanisms. Second, the ‘value’ of these parameters differs greatly between individual patients. As an example, while in some patients intratumoural inhibition of tumour-specific T cells will be the sole defect that needs to be addressed, in other patients the tumour may simply be insufficiently foreign to mount a clinically relevant T cell response in the first place. Because of this multifactorial nature of cancer–immune interactions, only combinations of biomarker assays can be expected to best reveal which aspect of cancer–immune interaction should be the focus in individual cases. In addition to describing which defects in cancer–immune interaction need to be remedied, the Cancer Immunogram should also be useful to discuss in which cases a given treatment option may not be needed. As a clinically relevant example, it is unclear whether in patients with an existing tumour-specific T cell response combined blockade of PD-1 and CTLA-4 will be preferable over single agent PD-1 blockade, both because of the toxicity associated with (combination) treatment and the very significant treatment costs of cancer immunotherapies.

## Session 2: Clinical immunotherapy and combinations

The first presentationof the afternoon ‘Immune checkpoint inhibitors and combination approaches: current status and future perspectives’ was by **Paolo Ascierto** from the National Tumour Institute in Naples, Italy. Until recently, most immunotherapeutic approaches used to combat cancer were ineffective, counteracted by the tumour’s ability to evade immune attack. However, extensive research has improved our understanding of tumour immunology and enabled the development of novel treatments that can harness the patient’s immune system and prevent immune escape. Over the last few years, through numerous clinical trials and real-world experience, we have accumulated a large amount of evidence regarding the potential for long-term survival with immunotherapy agents in various types of malignancy [1].

In 2011, the approval of ipilimumab, an anti-cytotoxic T-lymphocyte-associated protein (CTLA)-4 monoclonal antibody, for the treatment of metastatic melanoma represented the first step in a new era for immunotherapy. Subsequently, promising survival results have been achieved with the anti- programmed death (PD)-1 antibodies nivolumab and pembrolizumab in patients with various cancers that as well as advanced melanoma include non-small-cell lung cancer and renal cell cancer. These and other immunotherapy agents in development have been heralded as potential turning point in the treatment of cancer.

Given the numerous immune checkpoints that exist and the multiple mechanisms used by tumours to escape the immune system, targeting distinct checkpoint pathways using various combination approaches is an attractive therapeutic strategy with the potential to further enhance the antitumour immune response. Several clinical studies have shown that combining different immunotherapies can improve outcomes and more likely to offer additional clinical benefit compared with single-agent therapies. Immunotherapies are also being assessed in combination with other treatment modalities including chemotherapy, targeted agents, and radiation.

Although the research focus and main advances have to date been largely on melanoma, immunotherapies are being actively investigated in many other cancer types including those where treatment options for patients are limited. Currently ongoing and planned studies should help bring the benefits of these novel immunotherapies within the reach of patients with a wide range of cancers.

**Nicolle Rekers** from the Maastricht University Medical Centre–The Netherlands gave a presentation on ‘Combining radiotherapy and cancer immunotherapy: a paradigm shift?’

There is conclusive evidence that apart from its direct effects, radiotherapy (RT) can initiate an immune response. Previously, we have shown that the addition of L19-IL2 to RT was able to drastically increase the immune response, and that this combination therapy resulted in a long-lasting synergistic anti-tumour effect. Rekers hypothesised that tumour cells outside the radiation field would also be eliminated by this combination treatment (abscopal effect) and that tumours cannot be formed again after re-challenging cured animals (memory effect).  In fact RT+L19-IL2 was able to cure 100% of primary tumours and was associated with an increased percentage of CD8+ T cells inside these irradiated tumours. When a single RT dose of 15 Gy was combined with L19-IL2, 20% of the non-irradiated secondary tumours were cured. Interestingly, the non-irradiated tumours of mice treated with 15 Gy+L19-IL2 showed a significant (p < 0.01) increased percentage of CD4+ T cells compared to irradiated tumours. Fractionated radiotherapy combined with L19-IL2 caused a significant (p < 0.01) growth delay in the non-irradiated tumours, however, no secondary tumours were cured. Immunological analysis revealed an increase in PD-1 expression on T cells infiltrating these tumours, suggesting a more regulatory phenotype after fractionated radiotherapy compared with one single RT dose. Moreover, it is observed that new C51 tumours were not able to form in cured mice whereas 100% of the age-matched control mice formed tumours that reached established end-points within 17 days. Splenic T cells of these cured mice were associated with a high expression of CD127, a receptor associated with memory potential.

In conclusion the data show that RT+L19-IL2 causes anti-tumour immune effects also outside the radiation field, and that this effect is associated with an increase of CD4+ T cells. Cured mice were not able to form new tumours and have a high expression of CD127 on their T cells suggesting these cells have an immunological memory. This new treatment will be further investigated in a Phase I study for patients with an oligometastatic solid tumour (NCT02086721).

‘Cancer vaccines: still a viable approach?’ was the title of **Per Thor Straten’s** (University of Copenhagen) talk. He discussed the requirements for construction of this vaccine, the differences in preventive and therapeutic vaccinations and the associated immune responses, how and why cancer cells are targeted and the antigens recognised. A couple of examples of vaccine trials including a short sum-up of the FDA/EMA approved Sipuleu cell T were discussed along with the nature of clinical responses upon vaccination–and based on that–some discussion slides on whether therapeutic vaccination against cancer is still (if it ever was...) a viable approach....

Finally, the ‘Implications of modern immunotherapy on the organisation of healthcare in cancer centres’ was presented by **Ahmad Awada** (Institut Jules Bordet, Belgium).

This important therapeutic development of modern immunotherapy will surely have implications on the organisation of healthcare in cancer centres. Dr Awada discussed the reasons for this and these are summed up below:

- Potentially more patients will survive cancers. Medical problems of survivors should be managed optimally by cancer centres.
- There will be an increase in the number of patients treated, for long periods of time, at outpatient clinics.
- Predictive markers of sensitivity/resistance will be developed. Pathology and molecular biology departments should follow and implement these advances.
- Modern immunotherapy will have several organ-related adverse events. An multidisciplinary approach (including oncologists and internal medicine specialists) is needed to tackle these adverse events.
- Adoptive cell therapies are developing. Only specialised cancer institutes and university centres will be able to implement these approaches.
- Immunotherapy is beneficial in nearly all solid and some haematological malignancies. A ‘transversal’ immunotherapy unit for clinical and research aspects should be created in cancer centres.
- More and more patients are responding partially to these therapies. Consolidating this benefit with local therapy (surgery, radiofrequency, local radiotherapy...) should be developed and organised within the treating cancer centre.
